# Primary Vitreoretinal Lymphoma: Current Diagnostic Laboratory Tests and New Emerging Molecular Tools

**DOI:** 10.3390/curroncol29100543

**Published:** 2022-09-24

**Authors:** Beatrice Melli, Pietro Gentile, Davide Nicoli, Enrico Farnetti, Stefania Croci, Fabrizio Gozzi, Elena Bolletta, Luca De Simone, Francesca Sanguedolce, Andrea Palicelli, Maurizio Zizzo, Stefano Ricci, Fiorella Ilariucci, Cristiana Rossi, Alberto Cavazza, Stefano Ascani, Luca Cimino, Magda Zanelli

**Affiliations:** 1 Molecular Pathology, Azienda USL-IRCCS di Reggio Emilia, 42123 Reggio Emilia, Italy; 2 Department of Obstetrics and Gynaecology, Fertility Center, Azienda USL-IRCCS di Reggio Emilia, 42123 Reggio Emilia, Italy; 3 Clinical and Experimental Medicine PhD Program, University of Modena and Reggio Emilia, 41124 Modena, Italy; 4 Department of Surgical Sciences, Eye Clinic, University of Cagliari, 09124 Cagliari, Italy; 5 Clinical Immunology, Allergy and Advanced Biotechnologies Unit, Azienda USL-IRCCS di Reggio Emilia, 42123 Reggio Emilia, Italy; 6 Ocular Immunology Unit, Azienda USL-IRCCS di Reggio Emilia, 42123 Reggio Emilia, Italy; 7 Pathology Unit, Policlinico Riuniti, University of Foggia, 71122 Foggia, Italy; 8 Pathology Unit, Azienda USL-IRCCS di Reggio Emilia, 42123 Reggio Emilia, Italy; 9 Surgical Oncology Unit, Azienda USL-IRCCS di Reggio Emilia, 42123 Reggio Emilia, Italy; 10 Hematology Unit, Azienda USL-IRCCS di Reggio Emilia, 42123 Reggio Emilia, Italy; 11 Pathology Unit, Azienda Unità Sanitaria Locale ASL5 La Spezia, 19124 La Spezia, Italy; 12 Pathology Unit, Azienda Ospedaliera Santa Maria di Terni, University of Perugia, 05100 Terni, Italy; 13 Department of Surgery, Medicine, Dentistry and Morphological Sciences, University of Modena and Reggio Emilia, 41124 Modena, Italy

**Keywords:** lymphoma, vitreoretinal, IL-10, IGH, MYD88, NGS

## Abstract

Primary vitreoretinal lymphoma (PVRL), a rare aggressive malignancy primarily involving the retina and/or the vitreous, is a major diagnostic challenge for clinicians (who commonly misdiagnose it as chronic uveitis) as well as for pathologists (for biological and technical reasons). Delays in diagnosis and treatment are responsible for visual impairments and life-threatening consequences, usually related to central nervous system involvement. The identification of lymphoma cells in vitreous fluid, obtained by vitrectomy, is required for diagnosis. Of note, the scarcity of neoplastic cells in small volumes of vitreous sample, and the fragility of lymphoma cells with degenerative changes caused by previous steroid use for presumed uveitis makes diagnosis based on cytology plus immunophenotyping difficult. Interleukin levels, immunoglobulin heavy chain or T-cell receptor gene rearrangements, and MYD88 mutation are applied in combination with cytology to support diagnosis. We aim to describe the current laboratory technologies for PVRL diagnosis, focusing on the main issues that these methods have. In addition, new emerging diagnostic strategies, such as next-generation sequencing analysis, are discussed. The genetic profile of PVRL remains largely unexplored. Better knowledge of genetic alterations is critical for precision medicine interventions with target-based treatments of this lymphoma for which no standardised treatment protocol currently exists.

## 1. Introduction

Intraocular lymphomas (IOLs) include vitreoretinal lymphomas (VRLs) and primary uveal or choroidal lymphomas.

VRLs are further subdivided into primary VRLs and secondary VRLs, the latter deriving from systemic lymphomas.

Primary uveal or choroidal lymphomas are usually low-grade neoplasms and are frequently extranodal marginal zone lymphomas with very good outcomes, unlike primary vitreoretinal lymphomas (PVRLs) which are high-grade diseases with poor outcomes.

Secondary IOLs derive from ocular involvement by systemic lymphomas through haematogenous spread. Systemic lymphomas mainly disseminate to the uvea, due to its rich blood flow [1].

Over 95% of PVRL cases are diffuse large B cell lymphomas (DLBCLs), and only a minority are of T cell or natural killer (NK) cell origin. PVRLs are considered a subgroup of primary central nervous system lymphomas (PCNSLs), which primarily affect the retina with or without vitreous or optic nerve involvement [2]. In the new WHO-HAEM5 classification, the term ‘large B cell lymphomas (LBCLs) of immune-privileged sites’ has been introduced as an umbrella term that includes a group of aggressive B cell lymphomas that arise as primary tumours in the central nervous system (CNS), the vitreoretinal compartment, and the testes of immunocompetent patients. This new entity combines the previous entity of primary DLBCL of CNS with DLBCL of the vitreoretina and testis, previously included among DLBCL and NOS. These neoplasms involve immune sanctuaries and share biological, immunophenotypic and molecular features [3]. For simplicity, we will use the term PVRL to address neoplasms primarily involving the retina with or without vitreous or optic nerve involvement [2].

A PVRL may develop as an isolated entity or present before or simultaneously with a central nervous system (CNS) lymphoma [4]. After 16–24 months from PVRL diagnosis, 56–90% of patients develop CNS involvement [5]. Secondary vitreoretinal involvement may occur in the course of PCNSL, with 5–25% of PCNSL patients developing ocular involvement [5]. More rarely, spreading to the vitreous and/or the retina is reported to originate from systemic DLBCL [1].

PVRL is a real diagnostic challenge, often being clinically mistaken for a benign inflammatory ocular disease, and it is described as the most common uveitis masquerade syndrome [6]. The median time from the onset of symptoms to diagnosis ranges from 6 to 40 months [7,8].

The insidious clinical presentation as chronic/recurrent uveitis, combined with the transient response to steroids, which are lymphocytolytic, causes a delay in diagnosis and appropriate treatment, often resulting in a poor prognosis with high mortality if CNS is involved [9]. Local treatments, with or without systemic chemotherapy and radiotherapy, are the pillars of PVRL treatment, but due to disease rarity, no standardised protocol is currently available [10].

**Figure 1:**
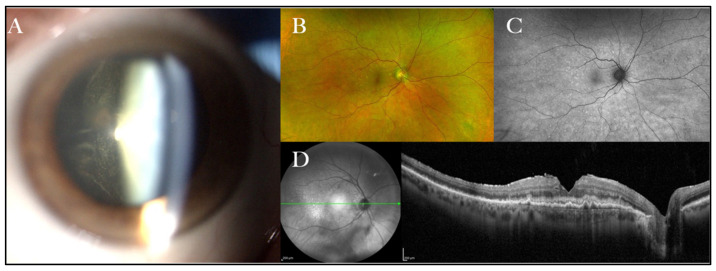
Common PVRL clinical features. (**A**) Sheets of tumour cells visible in the anterior vitreous; (**B**) colour fundus photography showing little round-shaped creamy retinal lesions; (**C**) fundus autofluorescence showing the same retinal lesions as (**B**) in a granular hyper-/hypo-autofluorescence pattern; (**D**) optical coherence tomography demonstrating malignant cell infiltration between the retinal pigment epithelium and the Bruch’s membrane at the macula (previously unpublished images).

An accurate ophthalmologic evaluation may reveal sheets or clumps of cells infiltrating the vitreous and multifocal creamy/white lesions in the outer retina, which are rather typical PVRL findings [11] (Figure 1).

**Figure 2:**
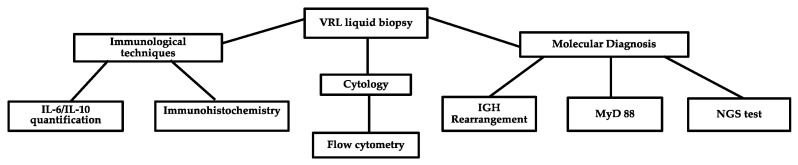
Flow chart of laboratory techniques used in PVRL diagnosis.

Diagnostic confirmation of the clinical suspicion is required through examination of vitreous fluid obtained by vitrectomy. The cytological analysis of vitreous fluid is considered the gold standard for PVRL diagnosis [12]. However, a limited volume of vitreous sample, low numbers of lymphoma cells, poor cellular preservation, and degenerative changes caused by steroid use for presumed uveitis, are the principal reasons for the rather variable (31–87.5%) and sometimes low diagnostic sensitivity of cytology alone [13]. Various laboratory techniques are employed to improve the diagnostic yield, including flow cytometry, immunocytochemistry, cytokine analyses, and molecular tests [2] (Figure 2).

An interleukin (IL)-10: IL-6 ratio > 1 is considered highly suspicious of lymphoma, as IL-10 is a growth factor for malignant B cells [14]. The sensitivity of cytokine testing in VRL diagnosis is elevated (93–94%) [14,15]. However, the use of steroids and certain systemic diseases may alter the cytokine profile, limiting the diagnostic power of this tool in some circumstances [16].

Molecular analyses can be performed even with a limited sample, being particularly useful on small amounts of vitreous fluid. Techniques such as DNA polymerase chain reaction (PCR) analysis require a low DNA quantity, allowing to identify clonal rearrangements of the immunoglobulin heavy (*IGH*) and light chains (*IGL*) or T cell receptor (*TCR*) gene rearrangements, as well as myeloid differentiation factor 88 (*MYD88*) mutation (mainly *MYD88 L265P* mutation) [17,18]. Despite clonality analysis being a valuable support for PVRL diagnosis, it can sometimes be either unsuccessful or misleading in the context of inflammatory conditions, with false-positive results due to the emergence of oligoclonal or clonal expansion.

Nowadays, *MYD88* mutational analysis is considered a valuable tool for diagnosis because of its high frequency in PVRL; however, as approximately 30% of PVRLs do not have an *MYD88* mutation, a negative result does not rule out PVRL diagnosis [2,17,18].

Young patients affected by VRL have some unique features, including lower *MYD88* rates, as recently reported by Liu et al. The authors found that the mutation rate of *MYD88* was significantly lower in young patients (50%) compared to elderly patients (91.3%); therefore, this result demonstrates that the diagnostic value of *MYD88* mutation is lower in young individuals [19].

In recent years, researchers have become increasingly interested in molecular diagnosis with next-generation sequencing (NGS) analysis, a massively parallel sequencing technology that offers ultra-high throughput, allowing to identify actionable genomic alterations.

Unlike PCNSL, little is known about the genetic profile of PVRL. Recently, a limited number of studies employing molecular profiling technologies including NGS analysis have been performed on PVRL, addressing the critical issue of how to develop new genetically targeted strategies for this aggressive disease [12,20,21,22,23].

This review analyses the main difficulties encountered in PVRL diagnosis with the laboratory techniques currently in use and in particular aims to focus on new molecular technologies, such as NGS analysis, which may enhance the diagnostic accuracy of this frequently misdiagnosed disease. A deep knowledge of PVRL’s genetic profile is also essential to define new target-based therapeutic options to improve patient outcomes.

## 2. Current Laboratory Techniques Used in PVRL Diagnosis and the Main Problems with the Different Methods

PVRL represents a diagnostic challenge for both clinicians and pathologists, and it is critical, for the patient’s life, to shorten the time between the onset of symptoms often mistaken for chronic uveitis and correct diagnosis. Different laboratory methods are in use to diagnose PVRL. The main currently employed techniques are described, highlighting the principal diagnostic issues with the different laboratory methods.

### 2.1. Cytology plus Immunohistochemistry

Cytological examination of vitreous fluid obtained through vitrectomy is considered the mainstay for PVRL diagnosis [2].

Decreasing the cut rate during vitrectomy is essential for obtaining a sufficient number of cells and further improving the diagnostic rate of cytological examination. Regarding this issue, Jiang et al. recommended a cut rate of 600 cpm or less during diagnostic vitrectomy to confirm VRL, because cell viability began to decrease at 600 cpm in the in vitro experiments [24].

The morphological assessment of the vitreous sample is complicated, requiring a pathologist with expertise in dealing with this kind of specimen.

The main difficulty is due to the small volume of vitreous sample, which often contains a low number of lymphoma cells. Lymphoma cells are known to be fragile and easily degenerate; therefore, the undiluted and refrigerated vitreous sample should be promptly transported within 1 h from the surgery room to the pathology laboratory and immediately processed.

Prior steroid treatments, performed in clinical suspicion of chronic uveitis, may not only delay diagnosis for a transient beneficial effect but compromise the morphological evaluation due to degenerative changes of lymphoma cells [25].

**Figure 3:**
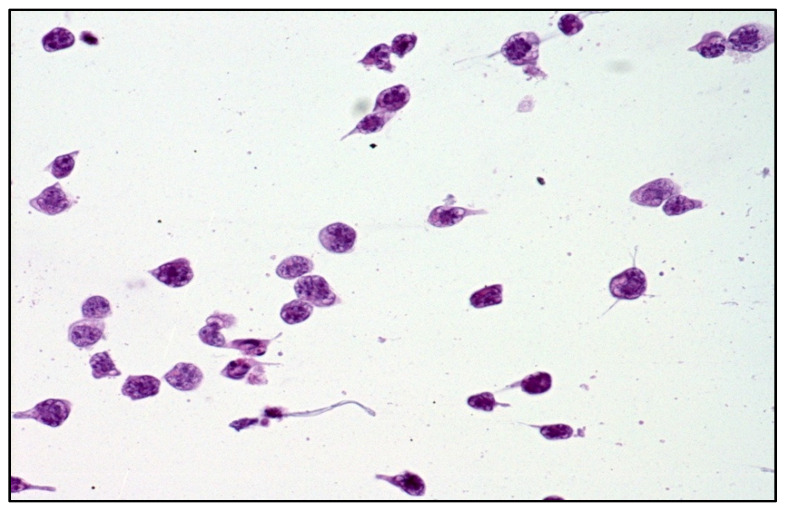
Cytology of vitreous sample with a discrete number of atypical lymphoid cells with a high nuclear–cytoplasmic ratio (haematoxylin and eosin, 200× magnification; original image from Dr M. Zanelli).

**Figure 4:**
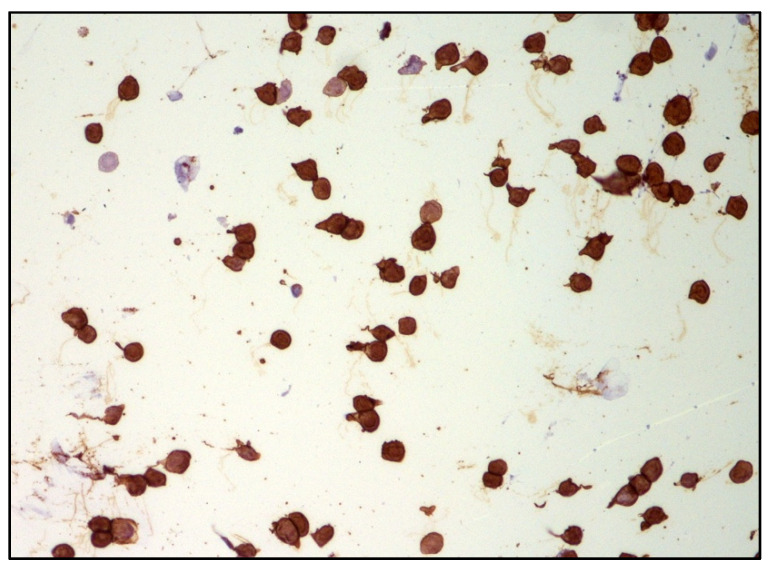
Immunohistochemistry performed on cytological sample of vitreous fluid: CD20 highlights the B cell phenotype of the majority of atypical cells (immunostaining; 200× magnification; original image from Dr M. Zanelli).

DLBCL cells are large-sized atypical elements with large irregular nuclei, evident nucleolus, and scanty cytoplasm (Figure 3 and Figure 4).

**Figure 5:**
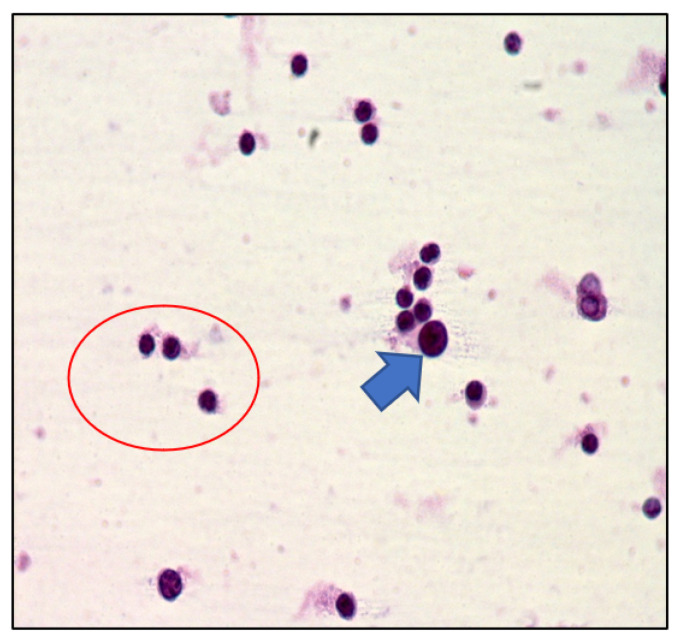
Cytology of vitreous sample with only rare large lymphoid cells and a discrete number of small lymphocytes (blue arrow pointing toward a large lymphoid cell; red circle highlighting small lymphoid cells) (haematoxylin and eosin, 200× magnification; original image from Dr M. Zanelli).

**Figure 6:**
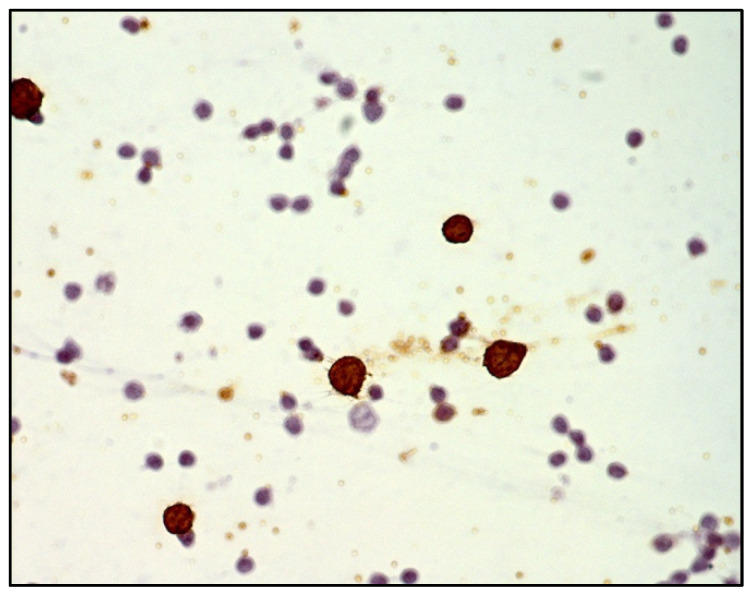
Immunohistochemistry performed on cytological sample of vitreous fluid showing only sparse CD20-positive atypical B cells (immunostaining; 200× magnification; original image from Dr M. Zanelli).

**Figure 7:**
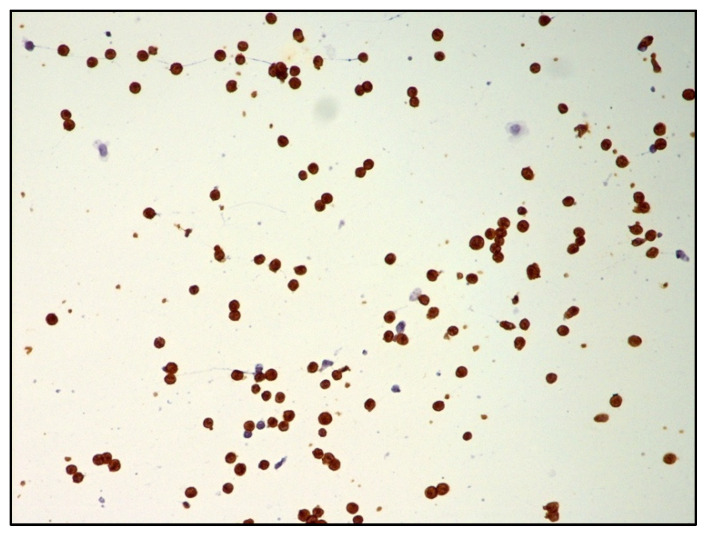
Immunohistochemistry performed on cytological sample of vitreous fluid showing numerous small-sized reactive CD3-positive T cells (immunostaining; 200× magnification; original image from Dr M. Zanelli).

However, the morphological assessment of vitreous fluid can be complicated by the presence of a background rich in inflammatory cells (T lymphocytes and histiocytes) admixed with only rare lymphoma B cells (Figure 5, Figure 6 and Figure 7).

Immunohistochemistry with B cell (CD20) and T cell markers (CD3) is often used in adjunct to cytology. However, the paucity and fragility of lymphoma cells often reduce the diagnostic power of immunophenotyping analysis. For the above-mentioned reasons, the sensitivity of cytology is variable (31–87.5%) [12,13] and further tests are needed in the case of a negative cytological result.

### 2.2. IL-10 and IL-6

The levels of both IL-10 and IL-6 may be measured in vitreous fluid using enzyme-linked immunosorbent assays (ELISA), multiplex bead-based assays, and cytometric bead array assays. IL-10 is a growth and differentiation factor for B lymphocytes, whereas IL-6 is produced by different types of cells, including inflammatory cells [15,26,27]. Unlike IL-6, which is a marker of inflammatory diseases, IL-10 is high in the intraocular fluid of PVRL patients, and an IL-10: IL-6 ratio over 1.0 is highly suggestive of lymphoma [2,14].

Recently, the ‘Interleukin Score for intraOcular Lymphoma Diagnosis’, or ISOLD, was developed [14]. Aqueous or vitreous IL-10 and IL-6 levels are inserted into a mathematical formula, resulting in a probability score for PVRL diagnosis. In the recent consensus recommendation paper for PVRL diagnosis by Carbonell et al., the IL-10 level or the IL-10:IL-6 ratio are considered useful parts of the diagnostic repertoire for PVRL diagnosis [2].

However, it needs to be underlined that cytokine production may be influenced by previous steroid or immunosuppressive therapies or certain systemic diseases, and hence, the diagnostic power of IL-10 to IL-6 ratio can be reduced [16]. Taking these limitations into consideration, cytokine analysis is currently considered a valuable adjunctive tool for screening patients suspected of PVRL.

### 2.3. Clonality Analyses

The majority of PVRLs are aggressive B cell lymphomas, mainly DLBCL, and a minority of cases are T cell lymphomas.

**Figure 8:**
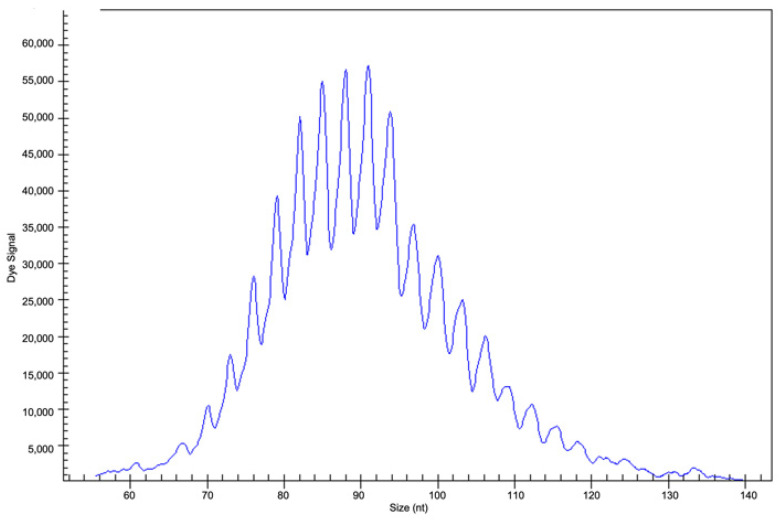
Fragment analysis by capillary electrophoresis: case analysed in clinical suspicion of PVRL, not confirmed by clonality analysis showing a polyclonal pattern suggestive of an inflammatory condition (previously unpublished image).

**Figure 9:**
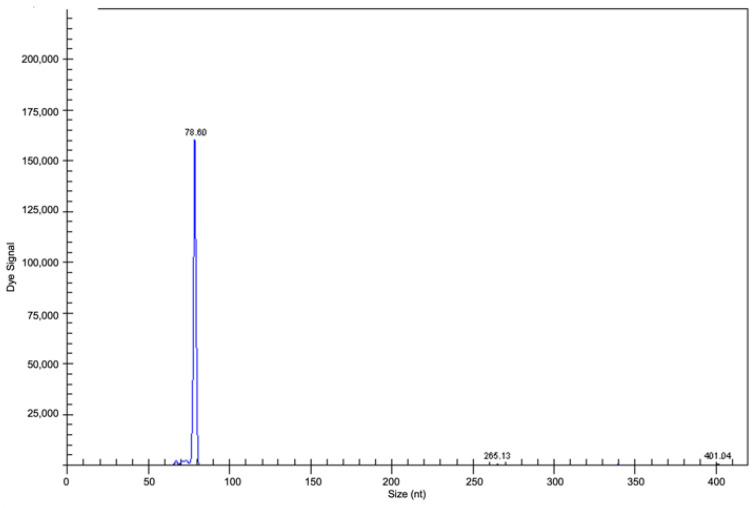
Fragment analysis by capillary electrophoresis: clonal gene rearrangement in CDR3 in the range of positivity 70–100 nt in a PVRL case (previously unpublished image).

The determination of clonality when evaluating *IGH* and *TCR* gene rearrangements is considered a valuable adjunct for lymphoma diagnosis. PCR analysis targeting rearranged *IG* genes gives multiple amplicons in the case of polyclonal cells, such as in inflammatory conditions (Figure 8), and a single amplicon if the cells are monoclonal and neoplastic, such as in lymphomas (Figure 9).

The accuracy of PVRL diagnosis is improved by the molecular analysis of DNA obtained by PCR, particularly in samples with low cellularity, poorly preserved neoplastic cells, or a prevalence of non-neoplastic T lymphocytes, in which cytology may give a negative result [16].

However, in vitreous samples, there is the potential risk of false negative or false positive results, even by clonality tests.

False negative results may occur because of the high frequency in PVRL of somatic hypermutation, potentially abrogating primer binding [1].

False positive results may be due to the detection of pseudoclonal/oligoclonal B cells by PCR due to the low cellularity of vitreous sample; this event may occur even in benign/inflammatory conditions, making the diagnosis of PVRL even more difficult [28,29].

### 2.4. MYD88 Mutation Analysis

The *MYD88* gene is on chromosome 3p22.2. The MYD88 protein, the gene product, is involved in signalling within the immune system. It is a cell membrane-associated protein acting as an adaptor molecule involved in Toll-like receptors (TLRs) and the interleukin-1 receptor (IL-1R) signalling pathway. Following a TLR stimulus, MYD88 activation causes intracellular signalling cascades, such as nuclear factor (NF)-kB activation, favouring the survival of tumour cells [30,31,32,33].

The change of adenine by guanine in the DNA sequence of *MYD88* results in the substitution in MYD88 protein of the amino acid lysine by proline at position 265; this determines the activation of B cells in various diseases [34,35].

DLBCLs arising in immune privileged sites are frequently associated with *MYD88* mutation, predominantly *L265P*. *MYD88-L265P* mutation is commonly associated with DLBCLs of activated B cell (ABC) phenotypes, such as PCNSL, in which the mutation is detected in approximately 75% of cases [36].

Bonzheim et al. retrospectively evaluated the frequency of *MYD88* mutation in PVRLs, analysing 75 vitrectomy specimens of 69 patients, and identified *MYD88* mutations in 69% of cases [17].

The high frequency of *MYD88* mutations, mainly *L265P*, identified in PVRL further supports the concept that PVRL and PCNSL represent the same disease [17]. Narasimhan et al. suggested that *MYD88* mutation analysis has a high diagnostic profile in terms of sensitivity, specificity, and accuracy and that detection of *MYD88* mutation significantly improves the diagnostic yield of vitrectomy samples [17,33].

**Figure 10:**
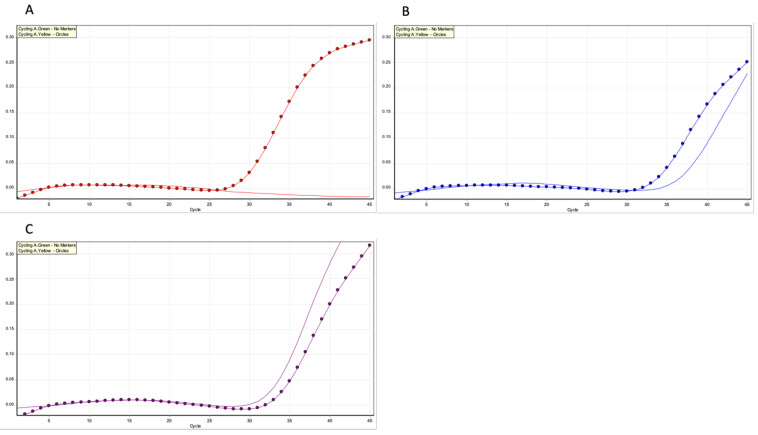
Real-time PCR analysis for *MYD88 L265P* mutation. Real-time PCR cycler with 2 channels (green, yellow); test performed through CORBETT/QIAGEN ROTOR-GENE RG-6000 REAL-TIME PCR. (**A**) Wild-type sample with one-channel amplifications; (**B**) mutated sample with two-channel amplification; (**C**) amplification of reference (previously unpublished image).

Real-time PCR is a variation of the standard PCR technique commonly used to quantify DNA or RNA in a sample. Using sequence-specific primers, the number of copies of a DNA or RNA sequence can be determined. By measuring the amount of amplified product at each stage during the PCR cycle, quantification is possible. The threshold of the real-time PCR reaction is the level of signal that reflects a statistically significant increase over the calculated baseline signal, as shown in the detection of *MYD88 L265P* mutation (Figure 10).

Several studies report that the PCR-based *IgH* rearrangement assay has some limitations, requiring a larger quantity of cells and having a higher limit of detection (10–20% of clonal B cell population) compared to *MYD88* mutation analysis, which may detect 5% or less of mutant cells [32,33,37,38,39].

### 2.5. Flow Cytometry

Flow cytometry is a technique used to identify phenotype cells by fluorescent antibodies or dyes. The use of flow cytometry in the diagnosis of lymphoma of B cell origin is based on the criteria that detecting the expression of either immunoglobulin kappa (IGK) or lambda (IGL) light chains may be suggestive of clonality.

In 1997, Davis et al. used flow cytometry for the first time in VRL diagnosis, with good results compared to cytology alone [13,40].

Despite being considered a valuable tool for VRL detection, with elevated sensitivity (82.4%) and specificity (100%) [41], it has to be taken into account that flow cytometry requires a large number of viable cells for diagnosis [37,42].

Hence, the low number of intact neoplastic cells in vitreous fluid, which is often combined with the presence of numerous inflammatory/reactive cells, unfortunately represents a critical limitation for the use of this technique in PVRL diagnosis [37,42].

## 3. New Emerging Diagnostic Molecular Tools

### 3.1. VRL Mutational Profile Analysed with High-Throughput Techniques: What Is Known So Far

In the current WHO classification, PCNSL is classified separately from conventional nodal DLBCL, as the disease is generally restricted to the CNS and its molecular profile is clearly distinct [43].

As previously mentioned, in the new WHO-HAEM5 classification, the term ‘LBCL of immune-privileged sites’ (CNS, testis and vitreoretina) has been introduced due to the common biological features of neoplasms arising at these sites [3].

PCNSL is usually a DLBCL of ABC phenotype and shows a high frequency of somatic hypermutation (SHM) of the rearranged *IGH* gene [44,45,46,47].

Molecular features often found in PCNSL are TLR and B cell receptors (BCR), signalling coactivation through *MYD88* and *CD79B* mutations, with the activation of the NF-κB pathway and homozygous losses of *CDKN2A,* leading to genomic instability and mechanisms of immune escape, namely the loss of the HLA locus on 6p21.33 and PD-L1/2 overexpression [46,47].

Unlike PCNSL, the mutational profile of both primary and secondary VRL remains insufficiently studied because of the rarity of the disease and the technical difficulties in obtaining an adequate sample of vitreous fluid.

To date, there are only a few studies evaluating VRL genetic alterations, often on a small number of cases [12,20,21,22,23].

In 2017, Cani et al. performed the first NGS-based analysis, using a panel of 126 genes, on 4 VRL cases [20]. Small volumes of diluted vitreous samples were used for NGS, obtaining sufficient DNA to analyse; therefore, the NGS approach did not compromise the volumes of non-diluted vitreous fluid necessary for cytology.

Genomic alterations, such as *MYD88* oncogene mutations and loss of tumour suppressors *CDKN2A* and *PTEN,* were identified by NGS. For the first time in VRL, *MYD88 S243N* was also found.

Interestingly, in the study by Cani et al., the NGS strategy detected genomic alterations in cases with previous false-negative cytological results which had caused a delay in diagnosis and treatment [20].

In 2022, Bonzheim et al. performed targeted NGS analysis on a large series of 34 vitreous fluids obtained by vitrectomy from 31 patients with either primary or secondary VRL [21].

The authors used a custom panel with genes frequently mutated in PCNSL. In a subset of cases, genome-wide copy number alterations (CNAs) were evaluated with the OncoScan technique.

The authors identified a high frequency of *MYD88* (74%), *PIM1* (71%) and *CD79B* (55%) mutations; other frequently found mutations were: *IGLL5* (52%), *TBL1XR1* (48%), and *ETV6* (45%). They also found frequent homozygous deletions of *9p21/CDKN2A* (75%). In the cases studied using the OncoScan platform, a high number of CNAs (18.6 CNAs per case) reflecting genomic instability were found.

According to Bonzheim et al., both primary and secondary VRL show a similar mutational pattern very close to PCNSL [21]. Similarly to PCNSL, VRL belongs to a specific cluster of DLBCL of ABC phenotype, named *MYD88*^mut^/*CD79B*^mut^ (MCD) or cluster 5 (C5), showing extranodal site involvement and possessing a poor outcome [48,49]. Similarly to two previous studies, Bonzheim et al. identified a lower rate of *CD79B* mutation in PVRL compared to PCNSL [22,23].

However, the previous studies noted a longer time to CNS involvement in wild-type *CD79B* cases, whereas Bonzheim et al. reported an incidence of *CD79B* mutation that was higher in PVRLs without CNS involvement (50%) than in cases with CNS involvement (33%), although the data were not statistically significant.

Secondary involvement of the vitreous by systemic DLBCL is generally rare, with the exception of testicular DLBCL, which frequently spreads to the CNS, vitreous and retina. DLBCL of the testis shares similarities with PCNSL, including the ABC phenotype and frequent *MYD88* and *CD79B* mutations [43].

Interestingly, in their series, Bonzheim et al. observed that about 50% of secondary VRL originated from systemic DLBCL rather than from PCNSL; however, these secondary VRL cases arising from systemic DLBCL showed a similar genetic profile [21]. According to the study by Bonzheim et al., secondary involvement of the vitreous, retinal layers and CNS depends on the biological characteristics of a particular DLBCL subset, specifically the MCD/C5 cluster, with a common molecular profile and the ability to involve immune-privileged extranodal sites, such as the vitreous, retina, CNS, and testis [21].

Recently, Balikov et al. made the first comparative NGS analysis of matched brain and vitreous samples in 2 patients presenting with PCNSL and VRL [12]. The authors showed that the two diseases were clonally related, having some alterations in common, such as *MYD88 L265P* mutations, and clonal *CDKN2A* deletions. Interestingly, subclonal alterations in *SETD2*, *BRCA2*, and *TERT* genes and broad chromosomal regions, revealed heterogeneity between the brain and the eyes, between the two eyes, and among different areas of PCNSL.

This NGS study supported the concept that PCNSL and VRL arising in the same individual have some similar genetic alterations, suggesting a common origin for these neoplasms. In addition, the two diseases also show distinct site-specific genetic alterations. Despite the limitations due to the small number of cases evaluated, the data provided by Balikov et al. have improved knowledge of the genetic relationships between these two related but site-distinct neoplasms [12].

Recently, Gu et al. performed NGS in cell free DNA (cfDNA) of the eyes with either VRL or inflammatory diseases and found that cfDNA sequencing of intraocular fluid can serve as the liquid biopsy to facilitate the diagnosis of VRL [50].

The NGS-based approach should not be used to replace conventional diagnostic techniques, but to increase diagnostic accuracy, overcoming difficulties which may be encountered with standard diagnostic methods. Additionally, by using the NGS approach, potentially targetable genetic alterations may be identified, giving patients the opportunity of new therapeutic strategies.

### 3.2. Quantification of microRNAs in Vitreous Specimens for Differential Diagnosis

MicroRNAs (miRNAs, miR) are small non-coding RNAs which can modulate the expression of multiple target RNAs post-transcriptionally. As well as being functional regulators, miRNAs can be biomarkers, and their quantification in biological samples can help in making differential diagnoses. MiRNAs have been profiled in vitreous samples (cell-free supernatants) from patients with PVRL by PCR array [51], real-time PCR [52], and miRNA oligo array [53]. MiR-155 has been detected at lower levels in PVRL than uveitis. However, the quantification of miR-155 in vitreous humour did not bring any benefits over the quantification of IL-6 and IL-10 ratios [51]. MiR-92, miR-19b and miR-21 have been found to be significantly upregulated in vitreous specimens from patients with PVRL. Receiver operating characteristic (ROC) curve analysis revealed that miR-92 had the best performance in discriminating between PVRL and vitritis [52]. Unsupervised clustering based on the expression of 2565 miRNAs allowed the separation of PVRL vitreous samples from macular hole/epiretinal membrane samples, and PVRL samples from uveitis samples. Ten miRNAs (miR-1273d, miR-133b, miR-146a-5p, miR-181-5p, miR-193b-3p, miR-221-3p, miR-326, miR-345-5p, miR-422a, miR-4655-3p) emerged up-regulated, both in the vitreous and serum, from patients with PVRL compared to controls. Three miRNAs (MiR-6513-3p, miR-138-2f-3p, miR-4445-3p) emerged up-regulated, both in the vitreous and serum, from patients with PVRL compared to those with uveitis. MiR-326 appeared the most promising for differential diagnosis between PVRL and controls, such as healthy patients, or patients with uveitis, macular holes, and epiretinal membranes; miR-6513-3p appeared the most promising for differential diagnosis between PVRL and uveitis with vitreous opacity [53].

Data regarding miRNA profiling are promising, but different top-ranking miRNAs for differential diagnosis are given in different papers. Further studies are thus needed to confirm these preliminary results. MiRNA quantification might be exploited as a new test in the diagnosis of PVRL. The advantage of quantifying miRNAs lies in their stability. In addition, miRNA quantification in vitreous humour supernatants leaves cells for molecular profiling.

## 4. Conclusions

Small volumes of vitreous sample, a low number of lymphoma cells (which are often admixed with inflammatory cells), poor cellular preservation, and false-negative or false-positive PCR-based clonality results, are the main technical factors of difficulty and delay in PVRL diagnosis.

It is critical for the prognosis of PVRL patients to reduce the time between the onset of symptoms often mistaken for uveitis and correct diagnosis. Therefore, there is an urgent need to overcome the difficulties encountered in PVRL diagnosis by conventional techniques.

So far, few studies on NGS analysis in PVRL have been performed. Despite the limited number of cases evaluated, NGS is currently emerging as a highly sensitive, adjunctive diagnostic method, which can be employed even on small quantities of vitreous fluid.

Additionally, an NGS-based approach may be fundamental in defining new therapeutic strategies based on the tumour genetic landscape. Further studies on a large number of cases are essential to improve the knowledge and prognosis of this rare, but aggressive, lymphoma.
